# Two long read-based genome assembly and annotation of polyploidy woody plants, *Hibiscus syriacus* L. using PacBio and Nanopore platforms

**DOI:** 10.1038/s41597-023-02631-z

**Published:** 2023-10-18

**Authors:** Hyunjin Koo, Gir-Won Lee, Seo-Rin Ko, Sangjin Go, Suk-Yoon Kwon, Yong-Min Kim, Ah-Young Shin

**Affiliations:** 1 Plant Systems Engineering Research Center, Korea Research Institute of Bioscience and Biotechnology (KRIBB), Daejeon, 34141 Republic of Korea; 2 SML Genetree Co. Ltd., Seoul, 05855 Republic of Korea; 3 Biosystems and Bioengineering Program, University of Science and Technology, Daejeon, 34113 Korea; 4 Department of Bioinformatics, KRIBB School of Bioscience, Korea University of Science and Technology (UST), Daejeon, 34141 Republic of Korea; 5 Digital Biotech Innovation Center, Korea Research Institute of Bioscience and Biotechnology (KRIBB), Daejeon, 34141 Republic of Korea

**Keywords:** Plant sciences, Plant genetics

## Abstract

Improvements in long read DNA sequencing and related techniques facilitated the generation of complex eukaryotic genomes. Despite these advances, the quality of constructed plant reference genomes remains relatively poor due to the large size of genomes, high content of repetitive sequences, and wide variety of ploidy. Here, we developed the *de novo* sequencing and assembly of high polyploid plant genome, *Hibiscus syriacus*, a flowering plant species of the Malvaceae family, using the Oxford Nanopore Technologies and Pacific Biosciences Sequel sequencing platforms. We investigated an efficient combination of high-quality and high-molecular-weight DNA isolation procedure and suitable assembler to achieve optimal results using long read sequencing data. We found that abundant ultra-long reads allow for large and complex polyploid plant genome assemblies with great recovery of repetitive sequences and error correction even at relatively low depth Nanopore sequencing data and polishing compared to previous studies. Collectively, our combination provides cost effective methods to improve genome continuity and quality compared to the previously reported reference genome by accessing highly repetitive regions. The application of this combination may enable genetic research and breeding of polyploid crops, thus leading to improvements in crop production.

## Background & Summary

Recent genome sequencing approaches, such as Pacific Biosciences Sequel (PacBio Sequel) and Oxford Nanopore Technologies (ONT), featuring long reads provide several advantages, which ultimately reduce additional sequencing costs and simplify preparation^1–4^. Such approaches hold promise for solving challenges associated with sequencing and assembling large, repetitive, and complex plant genomes through the production of large quantities of long reads to help bridge difficult regions in the genome^5,6^. Indeed, with notable improvements in long-read sequencing and related techniques, over 800 species of land plant genomes have been assembled recently^7,8^. Among these technologies, ONT led to a substantial improvement in plant genome contiguity and contig reduction. The wild tomato species *Solanum punnellii* (1.0 Gb) genome was assembled with a high contig N50 of 2.5 Mb using 135 Gb of ONT long-read data generated from 31 flow cells^9^. The highly heterozygous *Eucalyptus pauciflora* was sequenced and assembled using a combination of ONT long-read data (174×) and short-read Illumina data (228×) with five different assemblers. MaSuRCA generated the best assembly, which is 594.87 Mb in size, with a contig N50 of 3.23 Mb, and an estimated error rate of ∼0.006 errors per base^10^. Upon the advantages offered by long-read sequencing platforms, diverse plant genomes, including those of radish, oat, cotton, have been assembled at the chromosome level^11–13^.

Despite these advances, the quality of published sequences remains relatively poor^14^. The major barriers to plant genome sequencing and assembly are that plant genomes vary widely in size, have a high content of repetitive sequences, and exhibit a wide variety of ploidy^15–17^. Furthermore, isolation of high-quality, high-molecular-weight (HMW) DNA from plants poses a unique challenge due to rigid cell walls, co-purification of mitochondrial and chloroplast genomes, polysaccharides, and phenolic compounds that directly damage DNA, reducing sequencing yields^9,18–24^. Although various DNA extraction and library preparation protocols have been developed for different organisms or tissues of interest, obtaining reliable, high-quality yields from highly repetitive and polyploid plant genomes can be challenging. Furthermore, choosing among the many genome assemblers – such as Miniasm^25^, canu^26^, Flye^27^, wtdbg2^28^, SMARTdenovo^29^, Shasta^30^, NECAT^31^, and nextDenovo^32^ – can pose a barrier. NECAT relays a novel progressive, two-step error-correction algorithm with adaptive candidate-read selection for ONT raw reads^31^. NextDenovo is a string graph-based *de novo* assembler for long reads^32^. Flye relies on a repeat graph data structure that also tolerates more sequencing errors^27^. To date, few sequencing examples used more than three assemblers to construct large plant genomes, and available information on how to select *de novo* assembly tools or evaluate the quality of an assembled genome using ONT data is limited^33,34^.

To address this limitation, we report the *de novo* sequencing and assembly of representative hexaploid plant genome *Hibiscus syriacus* – a flowering plant species of the Malvaceae family – using two long read sequencing platforms: PacBio Sequel for *H. syriacus* cultivars cv. Baekdansim and ONT for cv. Gangneung. Due to the physically tough leaf tissues, which contain high levels of polysaccharides and phenolic compounds, it was challenging to isolate pure HMW DNA from *H. syriacus*. Therefore, we tested and optimized an efficient combination of intact HMW DNA isolation and sequencing using the ONT and PacBio. Additionally, we combined multiple assemblers to select the best genome assembly for the successful sequencing. To date, one *H. syriacus* genome was sequenced using short-read sequencing covering 92% of the genome with 1.7% gap sequences^35^. In the current study, the genome size (from 1.75 Gb in Illumina to 1.87 Gb in Nanopore) and contig N50 (from 140 kb to 8.1 Mb) were remarkably increased using ONT sequencing data (Table 1). Sanger sequencing evaluation revealed that the tandem repeat sequences missing from the Illumina-generated genome were successfully and accurately assembled into the new genomes with error correction by polishing. This *de novo* genome assembly strategy based on long-read ONT sequencing allows for construction of contiguous, improved-quality genomes. To date, genome assembly has been carried out for various species within the *Gossypium*, *Hibiscus*, and *Corchorus* genera of the Malvaceae family, ranging from scaffold level to chromosome level^11,36–39^. Our high-quality *H. syriacus* genomes provide an essential model to develop an effective strategy for polyploid plant genome assembly. These resources will provide valuable insight into functional genomics and evolutionary studies within the Malvaceae family.

**Table 1:** Statistics of Illumina and NECAT genome assembly of *H. syriacus* cv. Gangneung.

	Illumina^35^	ONT
Raw read coverage	122.8X (233.3 Gb)	63.4X (120.5 Gb)
Number of contigs	77,492	406
N50 (bp)	139,814	8,098,919
Average contig size	22,560	4,623,639
Number of Contigs >1 kb	34,103	405
Number of Contigs >100 kb	5,558	376
Number of Contigs >1 M	6	320
Number of Contigs >10 M	0	48
Total (Mb)	1,748	1,877

## Methods

### DNA preparation

High-molecular-weight (HMW) genomic DNA was extracted from leaf tissues of *H. syriacus* plants. We first isolate nuclei from *H. syriacus* plant cells using NIBM (10 mM Tris-HCl pH8.0, 10 mM EDTA pH8.0, 100 mM KCL, 0.5 M sucrose, 4 mM spermidine, 1 mM spermine, 0.15% ß-mercaptoethanol) buffer. From these intact nuclei, we successfully obtained high-quality genomic DNA using lysis buffer (50 mM Tris-HCl pH 7.5, 1.4 M NaCl, 20 mM EDTA pH 8.0, 0.5% SDS). Absorbance ratios to determine DNA quality ranged from 1.8 to 2.0 at A260/280 nm and from 2.0 to 2.2 at A260/230 nm using a Nanodrop spectrophotometer (Thermo Scientific). DNA size was assessed using a TapeStation system (Agilent). Most genomic DNA (gDNA) fragments were distributed between 10 and 100 kb.

### Library construction and sequencing

**Fig. 1:**
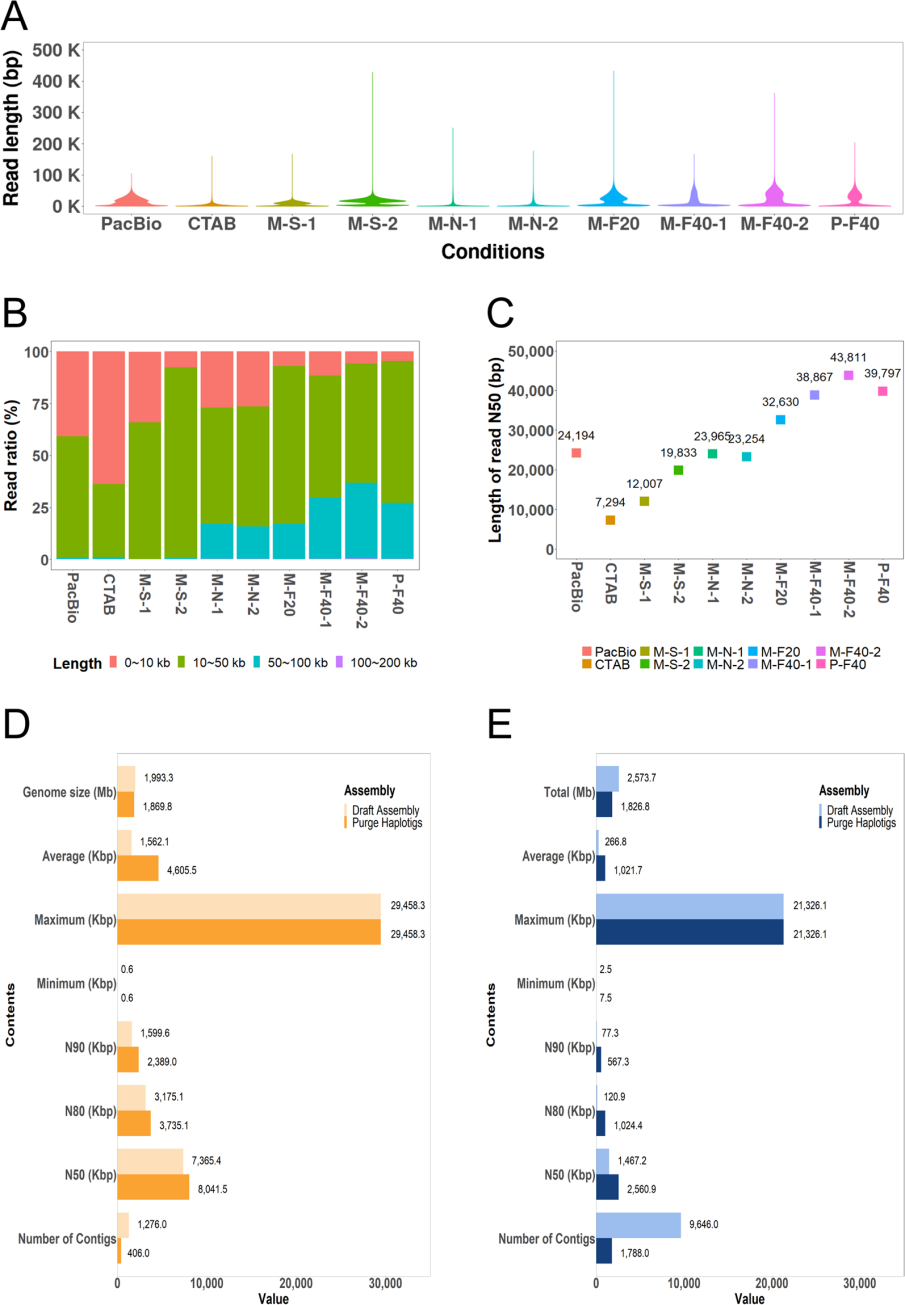
Distribution of raw reads from long read sequencing and genome assembly. (**A**) Read distributions from each sequencing batch. The *x*-axis indicates Nanopore sequencing batch, and the *y*-axis indicates read length. M, MinION; P, PromethION; S, sheared; N, non-sheared; F20, 20 kb size fractionation; F40, 40 kb size fractionation. (**B**) Read length ratio for each sequencing batch. The *x*-axis indicates ratio of read length, and the *y*-axis indicates sequencing batch. (**C**) Read N50 values for each sequencing batch. (**D,E**) Genome assembly improvements by Purge Haplotigs with ONT (**D**) and PacBio (**E**). The x-axis indicates value of parameters, and the y-axis indicates genome quality parameters.

To investigate optimal conditions for constructing a reference genome using long reads, we sequenced two *H. syriacus* cv. Baekdansim and Gangneung, using two sequencing platforms: PacBio Sequel and ONT. *H. syriacus* cv. Baekdansim, which has more complex genome structure, was sequenced using PacBio Sequel and cv. Gangneung was sequenced using ONT (Fig. 1). While the sequencing protocol using PacBio Sequel is well-defined, ONT sequencing for large and complex plant genomes requires optimization^36,40^. Therefore, using R9.4 MinION (M) or PromethION (P), we compared three library construction methods for ONT sequencing to optimize the final distribution of read lengths and total throughput: non-sheared gDNA (N), sheared gDNA (S), and size fractionation of non-sheared gDNA (F20 and F40). For shearing, 10 μg of pure HMW DNA was processed through a g‐TUBE (Covaris). For size-selective batches, a BluePippin system (Sage Science) was used with 10 μg of DNA without a shear step, followed by the selection of fragments >20 kb or >40 kb, and DNA recovery from the elution well (Fig. 1A,B). Sequencing libraries were prepared according to recommendations by ONT. The cetyltrimethylammonium bromide (CTAB) precipitation method, which eliminates polysaccharides from plant tissues^41^, was used as a control to evaluate the improvement of sequencing quality. The genomic DNA extracted using CTAB buffer underwent MinION sequencing after a DNA shearing step. MinION sequencing was performed as per the manufacturer’s guidelines using R9.4 SpotON Flow Cell (FLO-MIN106) and controlled using ONT MinKOW software. The final library batch was sequenced using PromethION (FLO-PRO002). Concerning high-quality DNA yields, longer N50 of read lengths were obtained with non-sheared DNA samples than with sheared DNA samples. DNA fractionation for library construction remarkably increased N50 of read lengths, with a maximum value of 43 kb (Fig. 1C).

### Genome assembly

**Fig. 2:**
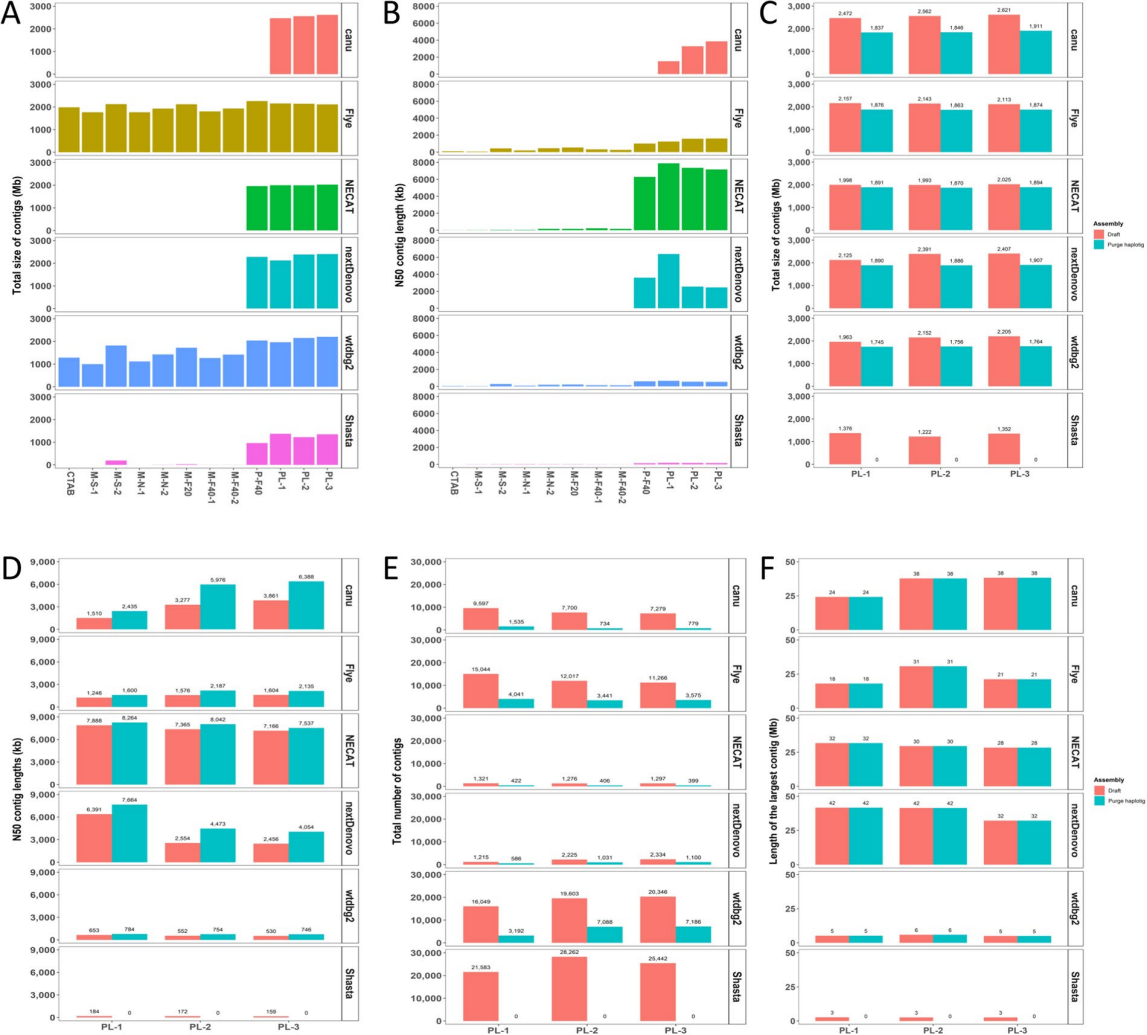
Assembly statistics for Nanopore sequencing. (**A**) Assembled genome size of each genome assembler. The x-axis indicates nanopore sequencing batch and the y-axis indicates total contig size. (**B**) Contig N50 of assembled genomes. The x-axis indicates nanopore sequencing batch and the y-axis indicates contig N50 length of the genome. (**C**) Assembled genome size for Draft and Purge Haplotigs-processed genomes. (**D**) Read N50 for Draft and Purge Haplotigs-processed genomes. (**E**) Number of contigs in the Draft and Purge Haplotigs-processed genomes. (**F**) Comparison of the longest contig lengths for Draft and Purge Haplotigs-processed genomes.

*De novo* assembly of *H. syriacus* cv. Baekdansim was performed with MECAT2, a time-efficient genome assembler known as the fast mapping, error-correction, and *de novo* assembly tool^42^ (Table 2). For cv. Gangneung genome assembly, we compared six *de novo* genome assemblers, canu (v.2.0), Flye (v.2.8.3), NECAT (v.0.0.1), nextDenovo, wtdbg2 (v2.5), and Shasta (v.0.1.0)) using individual sequencing data and combining datasets of MinION data (PL-1), size fraction data (PL-2), and whole data (PL-3) (Fig. 2A). Of the assemblers we tested, NECAT and nextDenovo performed significantly better than the others. Specifically, both showed a high level of contig N50 length over 8 Mb, whereas the other assemblers had lower contig N50 lengths (Fig. 2B). The comparison between PacBio and ONT showed that genome assembly using ONT generated a longer contig N50 and fewer contig numbers compared to PacBio (Table 2).

**Table 2:** Genome assembly statistics for the multiple reference genomes of *Hibiscus syriacus*.

	Baekdansim (PacBio)	Gangneung (ONT)
Assembler	MECAT	NECAT
Raw read coverage	74X (148.1 Gb)	63.4X (120.5 Gb)
Number of Contigs	1,788	406
N50 (bp)	2,560,905	8,098,919
N80 (bp)	1,024,377	3,747,792
N90 (bp)	567,339	2,397,205
Minimum (bp)	7,536	594
Maximum (bp)	21,326,069	29,519,541
Average (bp)	1,021,683.3	4,623,639
Protein-coding genes	88,414	88,573
Total (bp)	1,826,769,743	1,877,197,537

Regional duplication due to regional heterogeneity is one of the major barriers to genome assembly using long reads, especially in polyploid genomes that contain native duplicated chromosomes. These haplotype-fused contigs lead to larger genome assemblies and can be problematic for downstream analysis^43,44^. In our case, where the regional heterogeneity of sequenced reads was very high, and five genome assemblers assembled these regions into separate contigs. Canu and nextDenovo generated more haplotype-fused contigs compared to other genome assemblers (Fig. 2C,D). To remove heterozygous sequences from the assembled genome, reassignment of redundant contigs in the primary assembly was performed using Purge Haplotigs^44^ with read-depth cutoffs of 10 (low), 53 (mid), and 110 (high). After processing with Purge Haplotigs, the final *H. syriacus* cv. Gangneung genome size was smaller than predicted, and contig N50 lengths were improved with a remarkably fewer number of contigs (Fig. 2C–F). Remarkably higher content of haplotype-fused contigs was detected in cv. Baekdansim assembled using PacBio Sequel sequencing data compared to cv. Gangneung using ONT data (Fig. 1D,E). Then, the contigs of Gangneung were polished with ONT raw data thrice using nextPolish^45^ v.1.01 and two times with filtered Illumina reads used in the previous study^35^.

### Repetitive element annotation

**Fig. 3:**
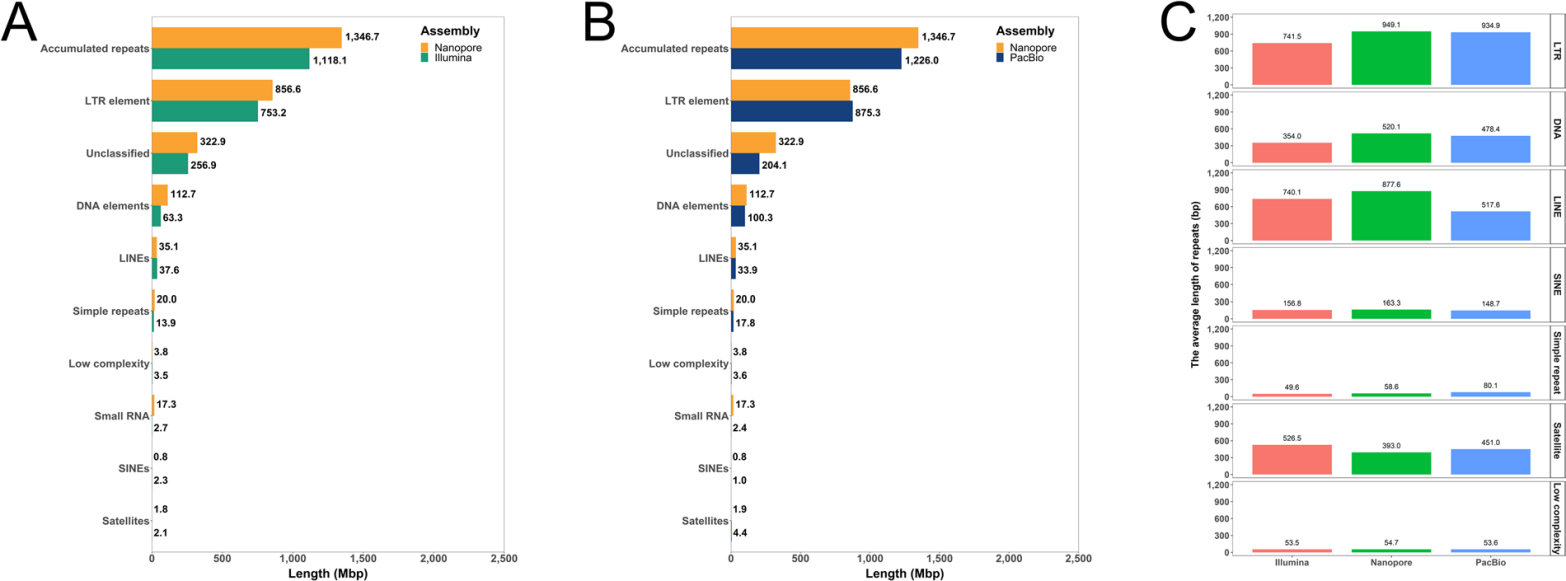
Repetitive element annotation. (**A**) Identification of repetitive sequences in Nanopore (orange) and Illumina (green). (**B**) Identification of repetitive sequences in Nanopore (orange) and PacBio (indigo). (**c**) The average repeat length distribution of each repetitive element types.

In general, repetitive sequences lead to genome assembly errors and automated gene annotation caused by frame shift of genes containing microsatellite sequences^46^. These barriers to genome assembly can be overcome using long-read sequencing technology. Thus, we compared the repeat content of previous and newly assembled genomes using repeat annotation analysis (Fig. 3). Repeat annotation was implemented using RepeatModeler and RepeatMasker as described previously^35^. After RepeatModeler was used to construct a repeat library with the assembled genomes, repeat annotation was performed using RepeatMasker (http://www.repeatmasker.org). We found that more repetitive sequences (about 215 Mb) of almost all categories were detected in the newly assembled genome using long-read sequencing (Fig. 3A). Further comparison of PacBio and ONT revealed higher repetitive contents and genome coverage in genome assembly using ONT (Fig. 3B). The average length of repetitive contents was also longer in ONT compared to Illumina sequencing (Fig. 3C).

### Genome annotation

Annotation was performed using the KOBIC annotation pipeline^35^ and consisted of repeat masking, mapping of different protein sequence sets, and *ab initio* gene prediction performed by AUGUSTUS v3.2.3^47^. Transcript assembly was performed by reference-based algorithm using HISAT2^48^ and StringTie^49^ with the assembled genome and RNA-Seq data in previous study^35^. The protein sequences of *Arabidopsis thaliana* (TAIR10, http://www.arabidopsis.org), *Theobroma cacao*^50^*, Gossypium raimondii*^36^, and *H. syriacus*^35^ were mapped using GeneWise v2.1^51^ to generate protein-based gene models for consensus modeling. AUGUSTUS was used for gene prediction. Subsequently, the predicted gene models from AUGUSTUS were validated using BLASTp with protein sequences from the four genomes (*T. cacao, G. raimondii, H. syriacus* and *A. thaliana*) as queries, and erratic gene models were filtered with a BLASTp cut-off value of query coverage ≥ 0.3. The assembled transcripts were validated using tBLASTn against the above-listed four protein sets and were filtered with query coverage ≥ 0.5 and subject coverage ≥ 0.3. The predicted gene models from GeneWise were also filtered using query coverage ≥ 0.3. The remaining gene models from GeneWise were reformatted to GFF3 format and were used to determine the consensus gene model via EVidenceModeler (EVM)^52^. EVM combines *ab initio* gene predictions with protein alignments into weighted consensus gene structures.

## Data Records

The assembled genome sequence of *H. syriacus* cv. Baekdansim has been deposited at NCBI, GenBank, under accession number VEPZ02000000^53^. All PacBio and Illumina raw read files are available through the NCBI Sequence Read Archive (SRA) with the identifier SRP193812^54^. The assembled genome sequence of *H. syriacus* cv. Gangneung has been deposited at NCBI, GenBank, under accession number JAUEMI000000000^55^. The Nanopore raw data are available through the NCBI Sequence Read Archive with the identifier SRP087036^56^. Both genome assembly and gene annotation results for *H. syriacus* cv. Gangneung and *H. syriacus* cv. Baekdansim are available at the online open access repository figshare database^57,58^.

## Technical Validation

### Distribution of raw reads and detection of potentially contaminated sequences

The distribution of reads indicated that the ratio of ultra-long reads (longer than 50 kb) in non-sheared pure HMW DNA with size fractionation conditions accounted for up to 36% of the total, which was significantly higher than other conditions, particularly compared to the general CTAB method (Fig. 1). Raw data read distributions showed that ONT generated a remarkably higher content of ultra-long reads, whereas PacBio Sequel generated a high content of relatively small long-read sizes ranging from 10 to 20 kb (Fig. 1A–C). Collectively, the high content of ultra-long reads might contribute to a more precise assembly of the cv. Gangneung genome by reducing the content of haplotype-fused contigs. The contig N50 read length and average contig length of both genome assemblies were improved by Purge Haplotigs (Fig. 1D,E).

In conventional DNA extraction methods, the step of removing organelles was absent, leading to relatively high contamination of organelle genomes as reported in the previous study^59^. To assess the proportion of organelle genomes, we mapped the ONT raw read data to the previously reported organelle genomes of *H. syriacus* and its related species. These results revealed that the prepared nuclear DNA had a notably small amount of unwanted organelle DNA contamination (Table 3). In plants, input data usually consist of 5–20% of unwanted organelle DNA reads, such as chloroplast and mitochondrial sequences^59^. These contaminant DNA reads led to over estimation of sequence coverage for genome assembly. Therefore, additional sequence data may be necessary to increase the quality of complex genome assembly. In our case, high-quality sequencing results were obtained with non-sheared, pure HMW DNA with an appropriate size fraction, resulting in a remarkably increased N50 of read length, maximum value of 43 kb (Fig. 1A,B). As unwanted organelle fragments were already filtered out, the *H. syriacus* genome could be fully completed even at the apparent 63.4× (120.5 Gb) coverage, which is relatively low depth compared to previous studies (Table 1).

**Table 3:** Investigation of organellar DNA contamination.

Organelle	Species	Accession ID	Genome size (bp)	Number of mapped read(%)
Chloroplast	*Gossypium arboreum*	NC_016712.1	160,230	0.3193
Chloroplast	*Gossypium hirsutum*	NC_007944.1	160,301	0.3193
Chloroplast	*Gossypium raimondii*	NC_016668.1	160,161	0.3193
Chloroplast	*Hibiscus syriacus*	NC_026909.1	161,019	0.3251
Mitochondrion	*Gossypium arboreum*	NC_035073.1	687,482	0.0039
Mitochondrion	*Gossypium hirsutum*	NC_027406.1	668,584	0.004
Mitochondrion	*Gossypium raimondii*	NC_029998.1	676,078	0.004
Mitochondrion	*Hibiscus cannabinus*	NC_035549.1	569,915	0.0052

### Quality assessment of the genome assembly

**Fig. 4:**
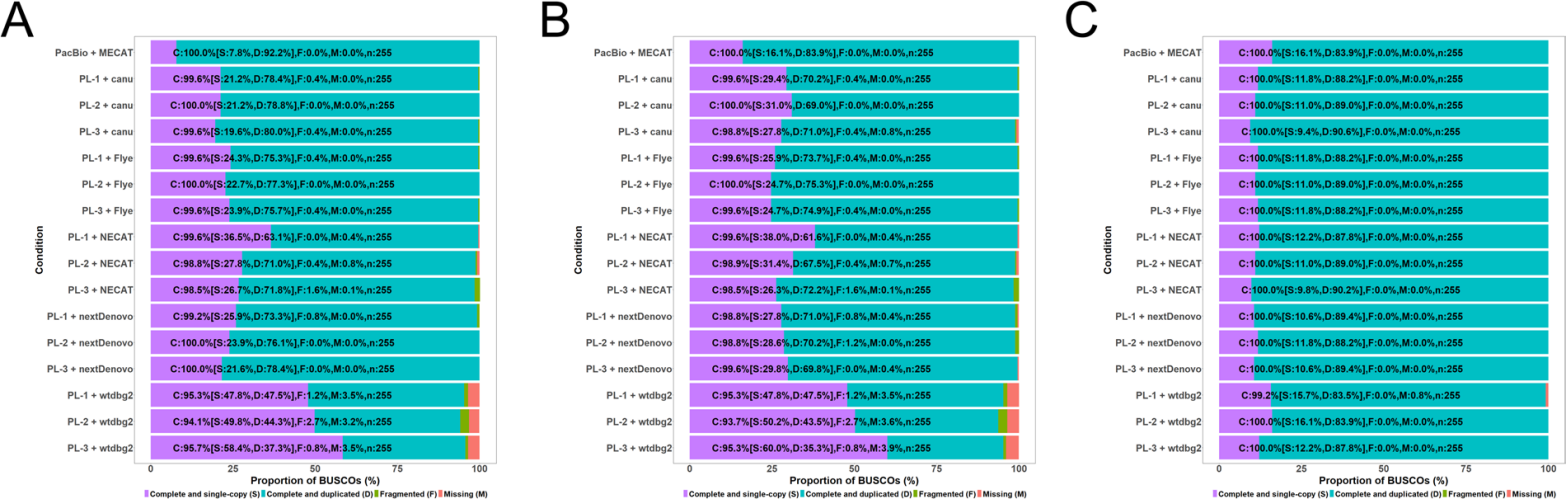
Quality assessment of the genome assembly. (**A**) Outputs from assemblies without further correction. (**B**) Assemblies corrected using Purge Haplotigs. (**C**) Assemblies corrected using NextPolish. The x-axis indicates the proportion of BUSCOs and the y-axis indicates individual genome assembly and their modified version. Purple shows the percentage of complete and single copy genes, the turquoise shows percentage of complete and duplicated genes, green shows the percentage of fragmented genes, and coral shows the percentage of of missing genes in the assemblies.

To assess genome assembly completeness, we performed BUSCO (Benchmarking Universal Single-Copy Orthologs) v5.3.2^60^ assessment of the assembled genomes using PacBio Sequel data and the combined ONT datasets (Fig. 4). Genome completeness ranged from 94% to 100% and revealed that wtdbg2 yielded the lowest completeness values (Fig. 4A). Relatively high levels of genome completeness were observed in canu, Flye, NECAT, and nextDenovo. This pattern was also seen in haplotype-fused contigs-removed genomes (Fig. 4B). The ratio of single- and duplicated-complete copy genes was decreased in polished genomes (Fig. 4C) as a result of recovering the collinearity of the duplicated gene by correcting the sequencing error using a polishing program. This correction step reduced fragmented and missing BUSCO genes and greatly facilitated genome completeness for all assemblies. Among them, genome assembly by NECAT using size fraction data showed a high contig N50 length with fewer contigs and high levels of genome assembly completeness, and was therefore selected for the next repeat sequence comparison.

### Genome assembly improvements by nanopore sequencing

**Fig. 5:**
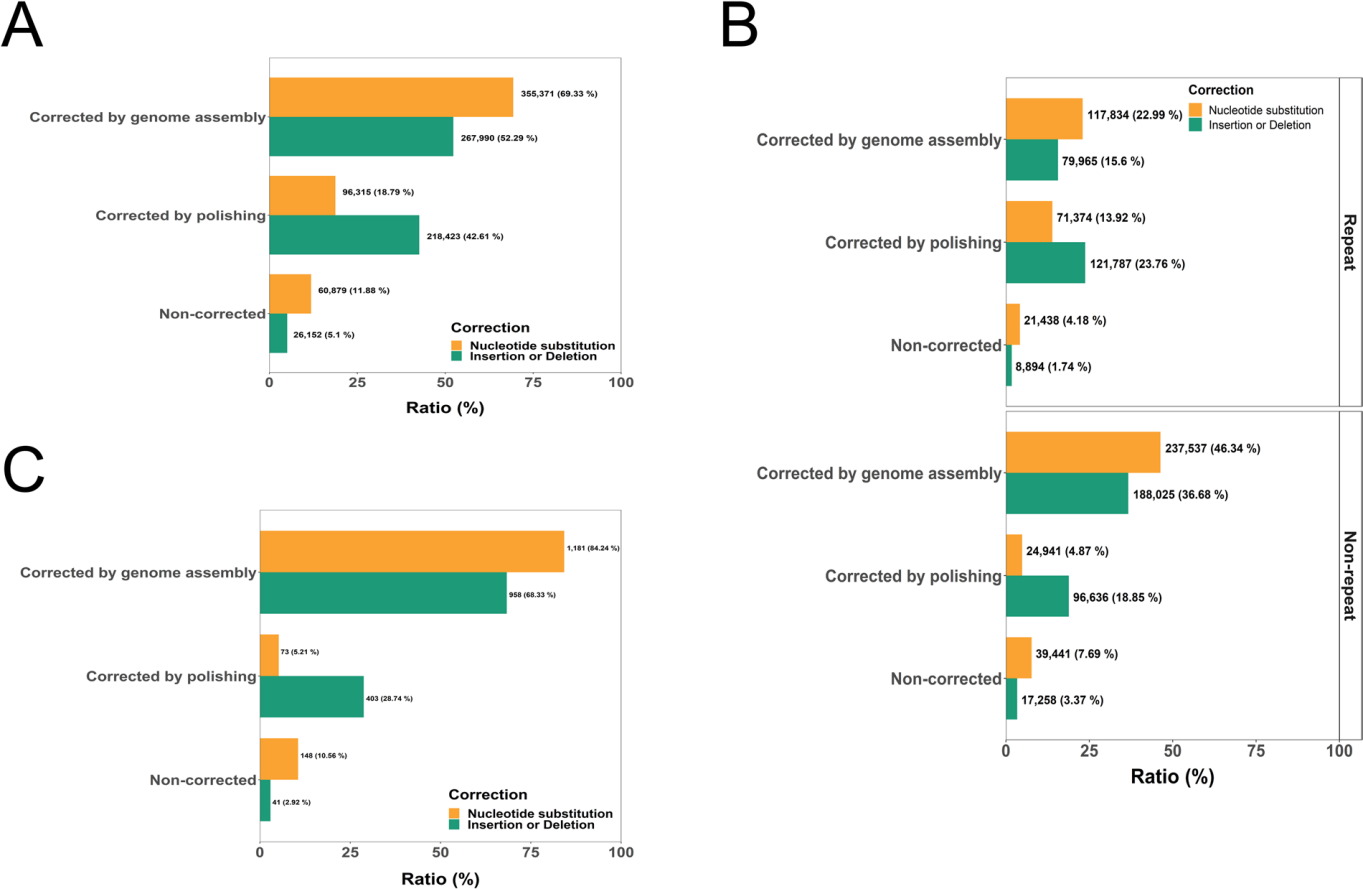
Genome assembly improvements by nanopore sequencing. (**A**) Identification of homopolymeric regions in draft genome assembly using NECAT, polished genome assembly, and Illumina genome assembly. (**B**) Homopolymer distribution in repetitive and non-repetitive sequences. (**C**) Identification of homopolymers in putative genic regions. Orange represents nucleotide substitutions and green represents nucleotide insertions or deletions.

Despite the development of an improved version of MinION^61^, long-read sequencing in previous studies showed a relatively low accuracy of 85–95%^62–64^. A common sequencing error is due to homopolymeric regions, or short repeat regions, which account for about half of all sequencing errors^61^. Despite vast improvements in raw error rate, assembled sequences still contain homopolymers, which are known to cause frameshift errors during gene annotation^65^. Polishing is one solution to correct these errors. To detect homopolymers and investigate their correction by polishing, each raw read batch was aligned to each version of draft genomes using Minimap2^66^ with default parameters for ONT sequencing and options (–secondary = no–sam-hit-only) to discard unmapped reads and perform secondary alignment as previously reported^61^ (Fig. 5). In total, 512,565 of 1,000 kb fragments containing homopolymeric regions were identified, and these homopolymers were mainly caused by sequencing errors in the newly assembled genome (Fig. 5A). Importantly, most sequencing errors were corrected during either the genome assembly or polishing step. A large proportion of nucleotide substitutions (69.33%) were corrected during genome assembly, whereas about half of the insertions or deletions were corrected by polishing. These homopolymers may arise due to low sequencing quality in the later stages of sequencing^67^. A higher proportion of homopolymers was detected in the non-repetitive sequences, including both genic and untranslated regions (UTR) (Fig. 5B,C).

**Fig. 6:**
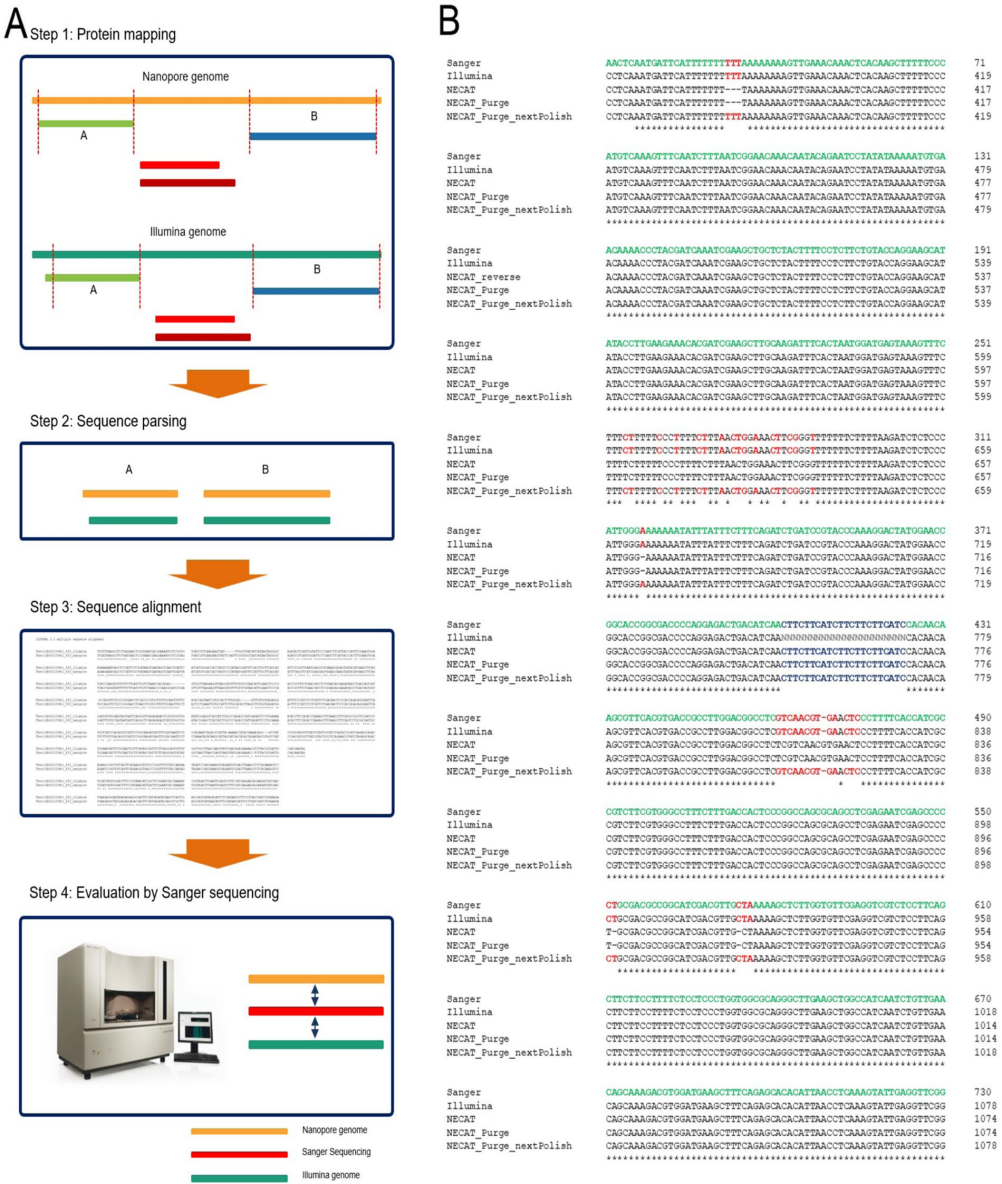
Identification of common genic regions between Illumina and nanopore assembly by NECAT. (**A**) Protein mapping were carried out using proteins of *Theobroma cacao* and *Gossypium raimondii* in step 1. In step 2, extraction of genome sequences from both genomes and sequence alignment were performed. Then, assembly errors by homopolymers were identified. In step 4, Sanger sequencing of sequences containing assembly errors were performed and multiple sequence alignments were carried out using Sanger sequencing results and genic sequences from Illumina and nanopore genomes. (**B**) Identification of assembly errors by homopolymers in genic regions. Sanger sequencing was used to identify the common genic region and multiple sequence alignment was performed to identify assembly errors by homopolymers in each version of genome assembly. Green, red, and blue represent Sanger sequencing results, assembly errors by homopolymers, and repetitive sequences missing in the Illumina genome, respectively.

We further investigated this type of error by evaluating common genic regions using tBlastN analysis with default parameters. For this analysis, we used proteins from the *Theobroma cacao* and *Gossypium raimondii* and Sanger sequencing (Fig. 6). The evaluation revealed tandem repeat sequences, which were missing from the previous genome, were successfully and accurately assembled in the new genomes (Fig. 6). Although nucleotide substitutions, deletions, or insertions were confirmed by Sanger sequencing, assembly errors due to this type of homopolymer were corrected during a polishing step (Figs. 5, 6). Collectively, these data suggest the application of Nanopore long-read sequencing technology has improved the construction of a reference plant genome, *H. syriacus*. Notably, about 215 Mb of repetitive sequences were incorporated into the newly assembled genome (Figs. 3A,B, 6).

## Data Availability

All software used for data processing were executed following the manual of the bioinformatic software cited above, and all commands used to assemble the genome are available in figshare^68^. If no detailed parameters are described for the software, the default parameters were used. Additionally, R codes used for figure construction are also available in figshare^68^.
